# Continuous glucose monitoring (CGM) in very low birth weight newborns needing parenteral nutrition: validation and glycemic percentiles.

**DOI:** 10.1186/s13052-018-0542-5

**Published:** 2018-08-22

**Authors:** Alessandro Perri, Lucia Giordano, Mirta Corsello, Francesca Priolo, Giovanni Vento, Enrico Zecca, Eloisa Tiberi

**Affiliations:** Division of Neonatology, Department of Pediatrics, University Hospital “A.Gemelli” Catholic University of the Sacred Heart, Rome, Italy

**Keywords:** Continuous glucose monitoring system, Very low birth weight, Glycemic percentiles, Neonatal intensive care unit, Parenteral nutrition, Preterm, Neonatal hyperglycemia, Neonatal hypoglycemia

## Abstract

**Background:**

Continuous glucose monitoring using subcutaneous sensors is useful in the management of glucose control in neonatal intensive care. We evaluated feasibility and reliability of a continuous glucose monitoring system in a population of very low birth weight neonates needing parenteral nutrition. Moreover, we presented percentiles of glycemia of the studied population.

**Methods:**

Very low birth weight neonates were enrolled within 24 h from birth. An ENLITE sensor connected to a continuous glucose monitoring system was inserted and maintained for at least 72 h. Data obtained with the continuous glucose monitoring system and with a glucometer were compared. Calibration was performed every 12 h.

**Results:**

Twenty-three patients (9 males) were included. Median gestational age was 28 weeks (range 23–30) and median birth weight was 860 g (range 500–1092). A total of 299 paired glucose values were obtained. Modified Clarke Error Grid criteria for clinical significance were met. 74 and 33 episodes of hypoglycemia and hyperglycemia were detected, respectively. 31,329 values of glycemia were analyzed and the percentiles calculated.

**Conclusions:**

This continuous glucose monitoring system is safe and accurate. It allows increasing the detection of hypo- and hyper-glycaemia episodes and it could be routinely used in the management of glucose infusion in very low birth weight neonates under total parenteral nutrition.

## Background

Very low birth weight (VLBW) neonates are at high risk of glycemic disorders. Neonates requiring intensive care have impaired glucose control and a wide fluctuation in blood glucose levels. Their developing brain is likely to be more susceptible to these metabolic insults. Hypoglycemia is associated with poor neurodevelopmental outcome [1–3] and hyperglycemia is correlated with increased mortality and morbidity in preterm infants [4]. Plasma glucose level detection represents the gold standard to diagnose these metabolic disorders, however, this method produces only punctual values and does not allow a real-time (RT) monitoring of glycaemia and its trends. This could explain the great deal of controversy over the values of normoglycemia in neonatal population and the paucity of proper diagnoses [5, 6]. Specifically, there is no consensus on the definition of “significant hypoglycemia” (blood glucose values requiring a medical treatment), and on the threshold of glucose concentration and the time needed to cause neurological damage [7, 8]. Continuous glucose monitoring (CGM) can be used to investigate glucose homeostasis during the neonatal period and some studies showed its use is safe and reliable [8–10]. Moreover, it is demonstrated that CGM can detect a significantly higher number of hypo/hyperglycemia episodes as compared to intermittent blood glucose measurement. A better management of glucose administration [11] can be obtained with a RT visualization of glycemic values in the new generation CGM system (CGMS) [12–14]. Recently, however, some authors expressed concerns about using CGM in the clinical setting [15]. In fact, they showed that the calibration methods of new generations CGM sensors are designed for higher glucose concentrations of children and adults, and not for neonates. Therefore, the routine usage of a CGMS with its specific sensor, could be proposed only after validation studies conducted in a neonatological setting. This study was conducted using RT Medtronic’s CGMS, a system previously tested by our research group in a population of neonates at risk of dysglycemia with a median gestational age (GA) of 32 weeks. Not many dysglicemic episodes were detected, but the CGMS demonstrated reliable in the normoglycemic range [12]. Only VLBW neonates fed by parenteral nutrition were included in this study, with the aim of testing the reliability of the Medtronic’s CGMS in this specific new setting. Babies had a lower mean gestational age and birth weight than in the previous study, and both hyper- and hypoglycemic episodes were detected during the study period. We also aimed at describing the distribution of glycemic values in the studied population. Such data is currently lacking in the scientific literature [16] for VLBW infant requiring parenteral nutrition, but it could be very useful to allow a proper modulation of the glucose infusion, thus preventing dysglycemic episodes, to reduce and optimize the need of insulin therapy, and hopefully to lead to the definition of a new protocol for a strict glycemic control in randomized controlled trials.

## Methods

### Population

All data were collected in the Neonatal Intensive Care Unit (NICU) of the Catholic University A. Gemelli Hospital between June 1st, 2016 and March 31st, 2017. This non-randomized feasibility study was conducted following the approval of our institutional review board. The study was aimed at testing the reliability of the Medtronic’s CGMS in a population of VLBW in parenteral nutrition and to investigate CGM data on the distribution of glycemic values in this specific population. Newborn infants were eligible for inclusion if they were VLBW and, accordingly to our internal protocols, a central line was positioned to administer parenteral nutrition. The criteria used in our unit to start parenteral nutrition, based on ESPGHAN, ESPEN and ESPR guidelines [17–19], were applied: Birth Weight (BW) ≤1250 g; 1250 g ≤ BW ≥ 1500 g associated with severe RDS (invasive mechanical ventilation; nCPAP and FI02 ≥ 0,3) or being small for gestational age with a documented prenatal history of severe placental insufficiency (umbilical doppler sonography assessing absent end diastolic (AED) or absent/reverse end diastolic (ARED) flow or brain sparing). Medtronic’s CGMS (Northridge, Calif., USA) is routinely used in our NICU to manage glucose infusion in all newborns at high risk of dysglycemia, and eligible neonates were enrolled in the study only if the instrument was available within 24 h from birth. Parenteral nutrition was administered following ESPGHAN, ESPEN, ESPR recommendations [17, 18]. The nutritional intake used in our unit is summarized in Table 1.

**Table 1:** Parenteral nutrition intake

	Day:	1	2	3	4	5	6	7	8	9	10
Glucose	g/kg/d	6,0	7,0	8,0	9,0	10,0	11,5	13,0	14,5	16,0	16,0
Lipid	g/kg/d	1,5	1,5	2,0	2,0	2,5	3,0	3,0	3,0	3,5	3,5
Amino acid	g/kg/d	2,5	3,0	3,0	3,5	3,5	3,5	4,0	4,0	4,0	4,0
Calcium	mg/kg/d	40	50	60	70	80	80	80	90	90	90
Phosforus	mg/kg/d		20	30	40	50	60	60	65	70	70
Magnesium	mg/kg/d			5	5	5	5	5	10	10	10
Sodium	mEq/kg/d				3	3	3	3	4	4	4
Chlorum	mEq/kg/d				3	3	3	3	4	4	4
Potassium	mEq/kg/d				3	3	3	3	4	4	4

Newborns with major congenital abnormalities at birth or with skin diseases were excluded. Informed consent was obtained from the parents, and infants enrolled were monitored with CGMS within the first 24 h of life for at least 72 h. In case of detachment or malfunction, the device was replaced (no more than once).

### CGMS

The CGMS is composed by the Enlite sensor, the Guardian transmitter and the VEO monitor. The new generation sensor has a cannula length of 8.75 mm. Other studies about CGMS reported the use of this device [11, 12, 14] but it is the first time, to our knowledge, that this CGMS is validated in this specific population of VLBW neonates fed by parenteral nutrition. The sensor of the CGMS used is equipped with a glucose-oxidase. This enzyme is used to detect the presence of glucose in the interstitial space. It makes the sensor generate an electrical current every 10 s. Data are then wirelessly transmitted to the monitor, that calculates and displays, every 5 min, the average of the currents measured. The calibration procedure permits the estimation and conversion into a blood glucose value from the measured current. The system was calibrated every 12 h, unless detected malfunctions required an additional calibration procedure. Calibrations were performed using point of care glucometer’s (Medtronic Stat Strip Xpress) glycemia value (GTX). The range of the interstitial glucose concentration values (mg/dl) is between 40 mg/dl (2.2 mmol/l) and 430 mg/dl (24 mmol/l). If CGM values do not fall within this range, they are expressed respectively as < 40 mg/dl or > 430 mg/dl. CGM data can be visualized in real time. The sensor was inserted, following a sterile procedure, in the lateral part of the thigh and a 33% glucose solution was orally administrated, if not sedated patients, before insertion. It was then connected to the transmitter. We collected all the patients’ personal and clinical data in a dedicated database. CGM data were downloaded via Care Link ™ software after sensor removal. Hypoglycaemia was defined as a glucose value ≤47 mg/dl [20], and hyperglycaemia as a glucose value ≥180 mg/dl. The monitor was programmed in order to give alarms when hyper- or hypoglycemia were detected. As a previous study conducted in our institution [12] demonstrated a good accuracy of this CGMS in detecting normoglycemia in a population of preterm newborns, glucose administration was based on values showed in RT by the monitor and reported by the nurses in the clinical chart, in specific time intervals (hourly or every 3h) according to our protocols. As there are concerns about the accuracy of CGMS in detecting hypoglycemia and hyperglycemia, a blood glucose test performed with the point of care glucometer was prescribed by the treating physician if episodes of hyper- or hypoglycemia were detected by the CGMS. An additional calibration of the instrument was performed if the CGMS glycemia value (CGMV) was inaccurate (|GTX-CGMV| > 20% CGMV). All the GTX obtained were compared with the correspondent CGMV. We preferred to use GTX (a point of care method) as a comparator, as it is most commonly used in clinical practice in the NICU as compared to laboratory enzymatic methods..

### Statistics

No formal sample size calculation was applied for this feasibility study. The comparison between two measuring techniques was assessed using the Bland-Altman plot and the Modified Clarke Error Grid (MCEG). MCEG definitions of neonatal hypoglycemia and hyperglycemia requiring a therapeutic treatment are used to evaluate the accuracy of a new tool, as compared to a standard method, for the determination of glycaemia in a clinical setting. MCEG shows the value generated by the monitoring system being tested along the ordinate axis, and the measurement of glucose as obtained with the reference technique along the abscissa axis. The MCEG identifies 5 areas (named Regions) with a different error in accuracy combined with the severity of clinical consequences. Region A: values within 20% of the reference sensor. Region B: values outside 20% of the reference sensor, but that would not lead to an inappropriate treatment. Region C: values leading to an unnecessary treatment. Region D: values indicating a potentially dangerous failure to detect hypo- or hyper-glycaemia. Region E: values that would confuse treatment of hypoglycemia for hyperglycemia, and vice-versa. Data obtained by the CGMS were also analyzed and their distribution was described. Estimates of the 5th, 10th, 50th, 90th and 95th percentiles were achieved. Secondary, number of episodes of hyper and hypoglycemia and their mean duration were obtained. The statistical analysis was performed using Xlstat, version 2014.5.03.

## Results

Twenty-three infants were enrolled, 9 males and 14 females, with a median birth weight (BW) of 860 g (range 500–1092 g) and a median gestational age of 28 weeks (range 23–30 weeks). Other population details are summarized in Table 2.

**Table 2:** Basic population details

Birth weight, median (IQR)	860 (500–1092)
Gestational age, median (IQR)	28 (23–30)
Sex ratio	9/14
AGA/SGA ratio	20/3
Delivery ratio CS/VAG	19/4
Apgar 1 min, mean (DS)	6 (2)
Apgar 5 min, mean (DS)	8 (1,6)
Tracheal tube 1 min (%)	0
Tracheal tube 5 min (%)	17,4
Early onset sepsis (%)	4,3
Late onset sepsis (%)	13
Treated with surfactant (%)	65,2
Mechanical ventilation after delivery (%)	82,6
Full enteral feeding during the observed period (n)	0

The sensor was well tolerated with an average (DS) duration of 112,7 (31,7) h. We collected 299 pairs of CGMV vs GTX measurements.

**Fig. 1:**
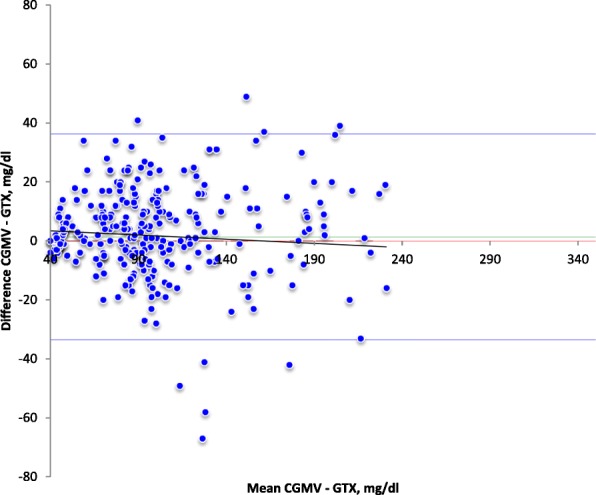
Bland Altman Analysis

Figure 1 reports the Bland Altman Analyses for glucose measurements. The mean (95% CI) difference was 1,4 (− 33,5 to 36,3) mg/dl.

**Fig. 2:**
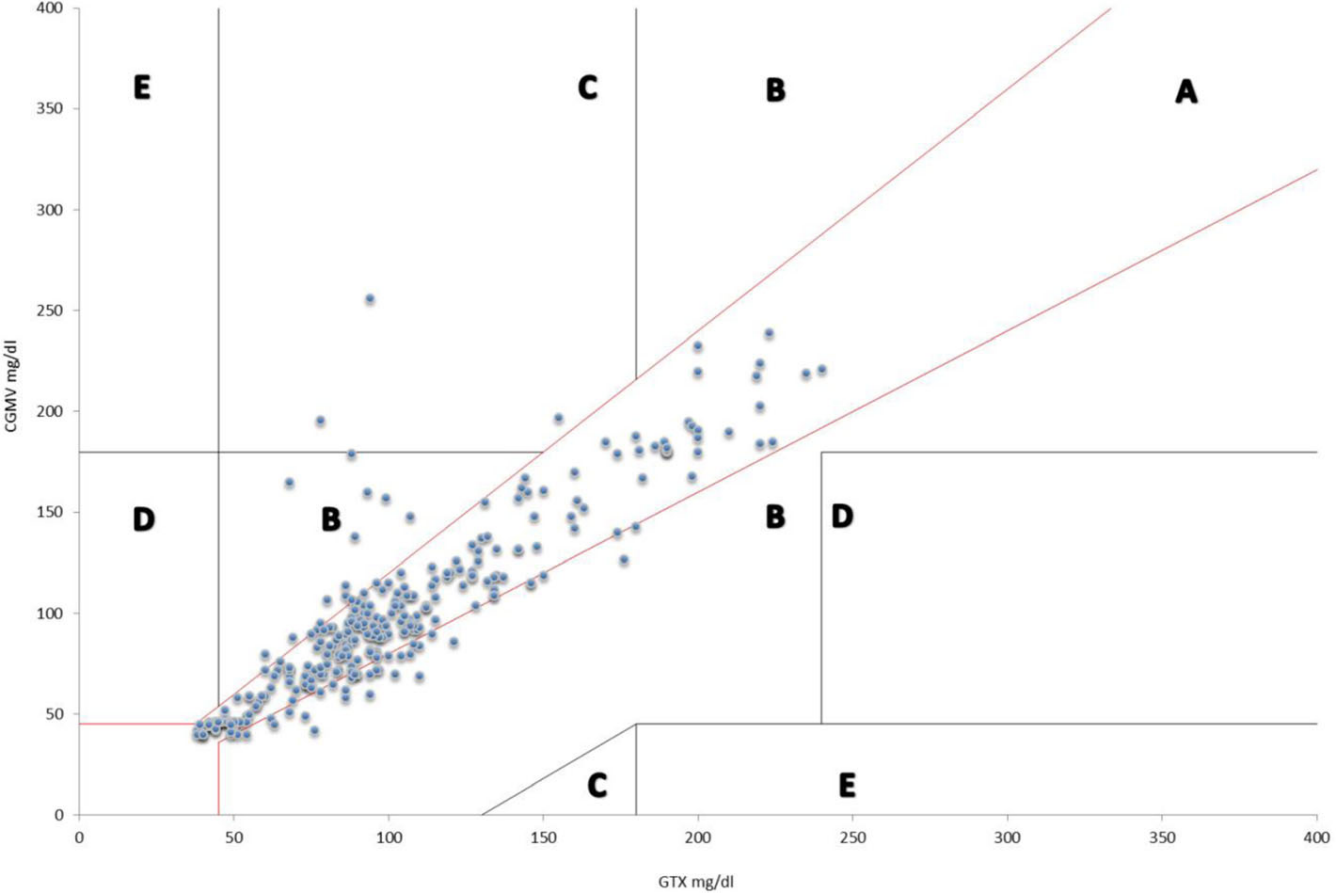
Modified Clarke Error Grid

Figure 2 reports the MCEG: 83,6% of measurements fall in region A, 15,4% of measurements fall in region B, and 1,0% of measurements in region D, without any value in region C or E. It is to be mentioned that glucose values obtained during the study did not fall under the lower limit detected by CGMS.

Seventy-four episodes of hypoglycemia and and 33 of hyperglycemia were detected; mean (DS) durations were 44 (48) and 96 min (130); 15 hypo- and 3 hyperglicemic CGMV turned out to be inaccurate at the blood sample analysis performed with point of care glucometer. 31,329 CGMV were obtained and analyzed.

Tables 3 and 4 present distribution and percentiles of glycemic values of the studied population.

**Table 3:** Distribution of glycemic values in the studied population

Lower Limit	Superior Limit	Frequency	Relative Frequency	Density
0	28	0	0,0000	0,0129
28	56	1807	0,0577	0,0795
56	84	9223	0,2944	0,2361
84	112	11,718	0,3740	0,3397
112	140	5402	0,1724	0,2368
140	168	2196	0,0701	0,0799
168	196	649	0,0207	0,0130
196	224	218	0,0070	0,0010
224	252	111	0,0035	0,0000
252	280	5	0,0002	0,0000

**Table 4:** Percentiles of glycemic values in the studied population

Percentiles	mg/dl
5°	54
10°	62
50°	93
90°	140
95°	159

## Discussion

CGM has a great potential for optimizing glycaemic control [21] and there is growing interest in using CGM devices in neonatal intensive care. However, as remarked in a recent review [15], CGM has a clear role in adult and child care but there are some concerns about its use in neonatology. This study evaluated feasibility and safety of the Medtronic’s CGMS in VLBW fed by parenteral nutrition. This CGMS proved to be safe to use in this population and it was well tolerated after the sensor’s insertion. Moreover, it didn’t interfere with nursing care and it showed an unexpected satisfaction by the neonatal staff. Furthermore, MCEG demonstrated the accuracy of the tool tested in the studied population. However, the data presented here show that this CGMS was inaccurate in some episodes of both hyper- and hypoglycemia, although all such inaccuracies by CGMV evidenced by GTX, fell in B region of MCEG, thus not leading to an inappropriate treatment. Some authors have published previous studies focused on CGM in neonates, suggesting the CGM as a useful instrument to be used in neonates requiring intensive care and in VLBW [9, 10, 22]. However, in all these studies both an old sensor and an old (not RT) continuous glucose monitoring system were used. More recently, the usefulness and reliability of RT-GCM was demonstrated in babies born at 32 weeks gestation who were at risk of hypoglycemia [23]. Moreover, a randomized controlled trial focused on VLBW babies, describes the CGM as a more effective tool in detecting and treating hypoglycemic episodes when compared to standard methods [11]. New glucose infusion protocols [13, 24], based on real time visualization of glycemic values, are incoming and will be proposed for newborns at high risk of hyper- or hypoglycemia. The main strengths of our study are the focus on a selected population at high risk of disglycemia and the use of a new generation sensor and a RT-CGM device. We also described the distribution of glycemic values of the studied population. At our knowledge it is the first time such an analysis has been performed. This distribution is very interesting, as in the future new glucose infusion rate protocols, based on the CGM usage, may be designed to tailor patient-based nutritional intake. Glycemic percentiles’ generation could allow to overcome the current limits of the “significant hypoglycemia” definition, correlating it to the glycemic trend rather than to punctual values. The main limitation of our study is the small size of the population. However, the observational design allowed to assess the efficacy and safety of the CGMS in this specific population. In conclusion, our results suggest that the presented CGM system with its sensor can be used, being both safe and accurate, in the management of glucose infusion and insulin therapy in VLBW neonates in total parenteral nutrition during the first week of life.

## Conclusions

Medtronic’s CGMS is safe and reliable in VLBW neonates fed by parenteral nutrition and is well tolerated after the sensor’s insertion. It doesn’t interfere with nursing care, as shown by the good satisfaction by the neonatal staff. Collecting glycemic values allowed to generate glycemic percentiles that could be useful to tailor patient-based glucose intakes. The data presented here are promising but a larger population of VLBW is needed in a randomized controlled clinical trial setting to properly define the place in therapy of CGMS.

## Abbreviations

AED: absent end diastolic
ARED: absent/reverse end diastolic
CGM: Continuous glucose monitoring
CGMS: continuous glucose monitoring system
CGMV: CGMS’s glycaemia value
GTX: glycaemia value
MCEG: Modified Clarke Error Grid
NICU: Neonatal Intensive Care Unit
RT: real time
VLBW: very low birth weight

## References

[1] Shang PW, Lu GZ, Sun X, Bian ZM, Shang ZY, Li J (2016). The influence of continuous glucose monitoring of high-risk neonate on guiding perinatal complications and one-year follow-up results. Eur Rev Med Pharmacol Sci.

[2] Rozance PJ, Hay WW (1997). Approaches to definition of neonatal hypoglycemia. Acta Paediatr Jpn.

[3] Rozance PJ, Hay WW (2006). Hypoglycemia in newborn infants: features associated with adverse outcomes. Biol Neonate.

[4] Rysavy MA, Marlow N, Doyle LW, Tyson JE, Serenius F, Iams JD, Stoll BJ, Barrington KJ, Bell EF (2016). Reporting outcomes of extremely preterm births. Pediatrics.

[5] Cornblath M, Hawdon JM, Williams AF, Aynsley-Green A, Ward-Platt MP, Schwartz R, Kalhan SC (2000). Controversies regarding definition of neonatal hypoglycemia: suggested operational thresholds. Pediatrics.

[6] Simmons R, Stanley C (2015). Neonatal hypoglycemia studies–is there a sweet story of success yet?. N Engl J Med.

[7] Hay WW, Raju TN, Higgins RD, Kalhan SC, Devaskar SU (2009). Knowledge gaps and research needs for understanding and treating neonatal hypoglycemia: workshop report from Eunice Kennedy Shriver National Institute of Child Health and Human Development. J Pediatr.

[8] Rozance PJ, Hay WW (2016). New approaches to management of neonatal hypoglycemia. Matern Health Neonatol Perinatol.

[9] Beardsall K, Vanhaesebrouck S, Ogilvy-Stuart AL, Vanhole C, VanWeissenbruch M, Midgley P (2013). Validation of the continuous glucose monitoring sensor in preterm infants. Arch Dis Child Fetal Neonatal Ed.

[10] Beardsall K, Ogilvy-Stuart AL, Ahluwalia J, Thompson M, Dunger DB (2005). The continuous glucose monitoring sensor in neonatal intensive care. Arch Dis Child Fetal Neonatal Ed.

[11] Uettwiller F, Chemin A, Bonnemaison E, Favrais G, Saliba E, Labarthe F. Real-time continuous glucose monitoring reduces the duration of hypoglycemia episodes: a randomized trial in very low birth weight neonates. PLoS One. 2015 Jan 15;10(1):e0116255. 10.1371/journal.pone.0116255. eCollection 2015 10.1371/journal.pone.0116255 PMC4295867 25590334

[12] Tiberi E, Cota F, Barone G, Perri A, Romano V, Iannotta R (2016). Continuous glucose monitoring in preterm infants: evaluation by a modified Clarke error grid. Ital J Pediatr.

[13] Galderisi A, Facchinetti A, Steil GM, Ortiz-Rubio P, Cavallin F, Tamborlane WV (2017). Continuous Glucose Monitoring in Very Preterm Infants: A Randomized Controlled Trial. Pediatrics.

[14] Szymońska I, Jagła M, Starzec K, Hrnciar K, Kwinta P (2015). The incidence of hyperglycaemia in very low birth weight preterm newborn. Result of a continuous glucose monitoaring study--preliminary report. Dev Period Med.

[15] McKinlay CJD, Chase JG, Dickson J, Harris DL, Alsweiler JM, Harding JE. Continuous glucose monitoring in neonates: a review. Matern Health Neonatol Perinatol. 2017;3:18. 10.1186/s40748-017-0055-z. 10.1186/s40748-017-0055-z PMC5644070 29051825

[16] Sinclair JC, Bottino M, Cowett RM (2011). Interventions for prevention of neonatal hyperglycemia in very low birth weight infants. Cochrane Database Syst Rev.

[17] Koletzko B, Goulet O, Hunt J, Krohn K, Shamir R, Parenteral Nutrition Guidelines Working Group (2005). Guidelines on Paediatric Parenteral Nutrition of the European Society of Paediatric Gastroenterology, Hepatology and Nutrition (ESPGHAN) and the European Society for Clinical Nutrition and Metabolism (ESPEN), Supported by the European Society of Paediatric Research (ESPR). J Pediatr Gastroenterol Nutr.

[18] Leaf A, Dorling J, Kempley S, McCormick K, Mannix P, Brocklehurst P (2009). ADEPT - abnormal Doppler enteral prescription trial. BMC Pediatr.

[19] Embleton ND, Simmer K (2014). Practice of parenteral nutrition in VLBW and ELBW infants. World Rev Nutr Diet.

[20] McKinlay CJ, Alsweiler JM, Ansell JM, Anstice NS, Chase JG, Gamble GD (2015). Neonatal glycemia and neurodevelopmental outcomes at 2 years. N Engl J Med.

[21] Chen C, Zhao XL, Li ZH, Zhu ZG, Qian SH, Flewitt AJ (2017). Current and Emerging Technology for Continuous Glucose Monitoring. Sensors (Basel).

[22] Iglesias Platas I, Thió Lluch M, Pociello Almiñana N, Morillo Palomo A, Iriondo Sanz M, Krauel VX (2009). Continuous glucose monitoring in infants of very low birth weight. Neonatology.

[23] Harris DL, Battin MR, Weston PJ, Harding JE (2010). Continuous glucose monitoring in newborn babies at risk of hypoglycemia. J Pediatr.

[24] Stensvold HJ, Lang AM, Strommen K, Abrahamsen TG, Ogland B, Pripp AH. Strictly controlled glucose infusion rates are associated with a reduced risk of hyperglycaemia in extremely low birth weight preterm infants. Acta Paediatr. 2017; 10.1111/apa.14164. 10.1111/apa.14164 29172239

